# Harnessing Pentameric Scaffold of Cholera Toxin B (CTB) for Design of Subvirion Recombinant Dengue Virus Vaccine

**DOI:** 10.3390/vaccines12010092

**Published:** 2024-01-17

**Authors:** Jemin Sung, Yucheol Cheong, Young-Seok Kim, Jina Ahn, Myung Hyun Sohn, Sanguine Byun, Baik-Lin Seong

**Affiliations:** 1 Department of Biotechnology, College of Life Science and Biotechnology, Yonsei University, Seoul 03722, Republic of Korea; jem9307@yonsei.ac.kr (J.S.); kys8726@naver.com (Y.-S.K.); 2 The Interdisciplinary Graduate Program in Integrative Biotechnology & Translational Medicine, Yonsei University, Incheon 21983, Republic of Korea; jina.ahnjina@gmail.com; 3 Department of Pediatrics, College of Medicine, Yonsei University, Seoul 03722, Republic of Korea; mhsohn@yuhs.ac; 4 POSTECH Biotech Center, Pohang University of Science and Technology (POSTECH), Pohang 37673, Republic of Korea; 5 Department of Microbiology and Immunology, College of Medicine, Yonsei University, Seoul 03722, Republic of Korea; 6 Vaccine Innovative Technology ALliance (VITAL)-Korea, Yonsei University, Seoul 03722, Republic of Korea

**Keywords:** dengue, cholera toxin B, recombinant protein, protein folding

## Abstract

Dengue virus is an enveloped virus with an icosahedral assembly of envelope proteins (E). The E proteins are arranged as a head-to-tail homodimer, and domain III (EDIII) is placed at the edge of the dimer, converging to a pentamer interface. For a structure-based approach, cholera toxin B (CTB) was harnessed as a structural scaffold for the five-fold symmetry of EDIII. Pivoted by an RNA-mediated chaperone for the protein folding and assembly, CTB-EDIII of dengue serotype 1 (DV1) was successfully produced as soluble pentamers in an *E. coli* host with a high yield of about 28 mg/L. Immunization of mice with CTB-DV1EDIII elicited increased levels of neutralizing antibodies against infectious viruses compared to the control group immunized with DV1EDIII without CTB fusion. IgG isotype switching into a balanced Th1/Th2 response was also observed, probably triggered by the intrinsic adjuvant activity of CTB. Confirming the immune-enhancing potential of CTB in stabilizing the pentamer assembly of EDIII, this study introduces a low-cost bacterial production platform designed to augment the soluble production of subunit vaccine candidates, particularly those targeting flaviviruses.

## 1. Introduction

Dengue virus (DENV), which belongs to the family *Flaviviridae* and is one of the most prevalent mosquito-borne viruses in the world [1], is estimated to cause approximately 60 million infections and 10,000 deaths annually [2]. Owing to global climate change, the risk area is rapidly increasing with the expansion of *Aedes aegypti* and *Aedes albopictus* habitats [3,4]. Infection with DENV may cause various clinical features, from mild flu-like symptoms (dengue fever, DF) to severe symptoms, such as hemorrhagic fever (dengue hemorrhagic fever, DHF) or hypovolemic shock (dengue shock syndrome, DSS), whereas secondary viral infection typically causes more severe symptoms [5]. Dengvaxia^®^ is the first licensed vaccine and has been approved in some impacted countries, such as Mexico, Brazil, Indonesia, Thailand, and Singapore [6]. Due to clinically identified safety issues of Dengvaxia^®^ despite its high neutralization capacity [7,8], there have been attempts to develop effective DENV vaccines using various production platforms: live-attenuated, inactivated, DNA, and recombinant protein subunit vaccines [9,10].

Among the three structural proteins of the dengue virion, capsid (C), membrane (M), and envelope (E) glycoproteins, E glycoprotein is involved in receptor binding, which presents a prime target for protective immune response [11]. The E glycoprotein consists of three domains: the central domain I (EDI) related to a conformational change in low pH conditions, domain II (EDII), which elicits cross-reactive antibodies that can cause antibody-dependent enhancement (ADE), and domain III (EDIII), which can induce serotype-specific neutralizing antibodies [9,12,13,14]. The dengue virion arranges into an imperfect icosahedral structure and consists of 180 E proteins that assemble into head-to-tail homologous dimers with elongated shapes. EDIII resides at the end of the E dimer and is positioned along the icosahedral five-fold axis and converges to the pentameric interface [12,15,16]. EDIII maintains the pentameric interface, not only in the mature virion, but even after low pH-induced conformational change during cell fusion [12]. Thus, the present strategy of assembly and display pentameric EDIII-mimicking infectious virions can be utilized to target both pre- and post-cell fusion stages.

Recombinant protein vaccines can be produced from various expression hosts, such as *Escherichia coli*, yeast, insect cells, plants, and mammalian cells. Soluble recombinant antigens tend to induce a weak immune response and therefore should be combined with adjuvants as additional vaccine components or assembled in particulate form to enhance vaccine efficacy [17,18]. For instance, monomeric antigens should be assembled into highly repetitive structures, effectively enlarging the antigen size; hence, they are more recognizable by immune cells [19,20,21]. If the assembly process is pivoted by adjuvants with self-assembling characteristics, the resulting vaccine formulation would be highly effective in eliciting protective immune responses.

Here, we present a novel pentameric assembly platform for viral antigens, using naturally occurring mucosal adjuvants. Our approach was to emulate the five-fold symmetry of DENV EDIII using the cholera toxin B subunit (CTB) of the AB5-type toxin family [16,22]. As a non-toxic component (B5) of the AB5-type holo-toxin family, CTB provides a pentamer scaffold for the assembly of the EDIII domain emulating the native five-fold axis [18,23]. Furthermore, CTB exhibits strong adjuvant activity owing to its ability to bind specific receptors (monosialotetrahexosylganglioside; GM1) [23]. Here, we hypothesized that genetic fusion between CTB and DENV EDIII could facilitate pentameric self-assembly and display of EDIII protective epitopes. In practice, however, combining the antigen of interest with a mucosal protein adjuvant into a chimeric antigen is technically very difficult because of the kinetic complexities in the folding pathway. For example, chimeric CTB proteins are highly refractory to soluble expression in bacterial hosts [22,24]. This problem can be partially bypassed by the chemical refolding of insoluble inclusion bodies, which inevitably results in either hetero-pentameric or homo-pentameric structures in low yields [24,25]. Thus, developing an effective recombinant vaccine using a microbial system remains a bottleneck for the delivery of low-cost vaccines in the endemic areas of low- and middle-income countries (LMICs) [26,27]. Using a novel RNA-based chaperone (Chaperna) system [28], we successfully demonstrated the pentameric assembly of soluble chimeric CTB-DV1EDIII in an *E. coli* expression system with enhanced neutralizing antibody titers compared to the monomeric antigen against the DENV. As a proof-of-principle affordable DENV vaccine for LMICs, this approach could serve as a widely applicable vaccine design platform for dengue and other flaviviruses of the icosahedron family.

## 2. Materials and Methods

### 2.1. Expression Vector and Mutagenesis

The expression vector pGE-hRID (the N-terminal domain of human lysyl-tRNA synthetase)(3) has been previously described [29]. The dengue virus 1 EDIII (DV1EDIII) gene (Lys_227_–Gly_327_, DenKor-07 strain) was digested with the restriction enzymes *Sal*I and *Hind*III and inserted into the pGE-hRID(3) vector to generate hRID-DV1EDIII. CTB was designed in the truncated form of its leader sequence (Thr_22_–Asn_124_, GenBank accession number HAS3667523) to improve its solubility [24]. The encoding gene was cleaved with *Kpn*I and *Sal*I and inserted between hRID and DV1EDIII in the pGE-hRID(3) vector to generate hRID-CTB-DV1EDIII. Two CTB mutants, the pentamer formation-defective mutant C9S and the GM1-binding defective mutant Y12D [30], were also inserted into the pGE-hRID(3) vector in the same way, corresponding to hRID-mCTB(C9S)-DV1EDIII and hRID-mCTB(Y12D)-DV1EDIII, respectively. As a control, a direct expression construct without the hRID fusion partner was also generated. The vector into which CTB and DV1EDIII were inserted was cut using *Nde*I and *Kpn*I to remove hRID, generating CTB-DV1EDIII.

### 2.2. Protein Expression and Purification

The *E. coli* strain SHuffle^®^ T7 (New England Biolabs, Inc., Ipswich, MA, USA) was transformed with each expression vector. The cells were cultured in 500 mL of LB medium at 30 °C with ampicillin (50 μg/mL) until the optical density at 600 nm (OD_600_) reached 0.8. Protein expression was initiated with 1 mM isopropyl β-D-1-thiogalactopyranoside (IPTG) and incubated at 16 °C overnight. The cells were separated from the medium by centrifugation (3000× *g* for 20 min), and the harvested cells were lysed by sonication in a lysis buffer (50 mM Tris-HCl [pH 7.5], 300 mM NaCl, 10% glycerol, 2 mM β-mercaptoethanol). The cell lysates were filtered through a polyethersulfone syringe filter with a 0.45 μm pore size (Hyundai Micro Co., Ltd., Seoul, Republic of Korea) after centrifugation (16,000× *g* for 10 min) and purified by Ni-affinity chromatography (Bio-Scale™ Mini Nuvia™ IMAC Cartridges; Bio-Rad Laboratories, Inc., Hercules, CA, USA). Purified samples were concentrated using Centriprep™ (Merck Millipore Ltd., County Cork, Ireland) and treated with AcTEV™ Protease (Invitrogen, Carlsbad, CA, USA) to cleave hRID at 4 °C overnight. The multimeric status of each sample was analyzed by native SDS-PAGE without dithiothreitol (DTT)/heat treatment and size-exclusion chromatography (SEC) using a Superdex™ 200 Increase 10/300 GL column (GE Healthcare, Uppsala, Sweden). A functional assay of pentameric CTB was conducted by monosialoganglioside GM1 binding.

### 2.3. GM1 Binding Assay

A GM1 binding assay was conducted to confirm whether samples from SEC form pentamers and to determine the specific binding affinity of samples for GM1 as the receptor for cholera toxin. Nunc 96-well microtiter immunoplates (Thermo Fisher Scientific, Rochester, NY, USA) were coated with 300 ng/well GM1 ganglioside (Sigma, Burlington, MA, USA) diluted in carbonate-bicarbonate buffer (pH 9.6; Sigma, Burlington, MA, USA) and incubated at 4 °C overnight. The plates underwent three washes with PBST (phosphate-buffered saline with 0.05% Tween 20) and were blocked with 1% BSA (bovine serum albumin) in PBST for 3 h at room temperature. The recombinant protein samples were serially diluted twofold from 300 ng first in wells with PBST and then applied to GM1-coated wells for 4 h at room temperature. Primary anti-Penta-His antibody (100 μL/well; Qiagen, Tegelen, Netherlands) and secondary antibody conjugated with HRP (100 μL/well; Bethyl Laboratories) were added for 1 h at room temperature. The plates underwent three washes with PBST and were incubated for 30 min in the dark after the addition of TMB (3, 3′, 5, 5′ tetramethylbenzidine (TMB) substrate reagent (BD Biosciences, Franklin Lakes, NJ, USA). After 30 min, 2 N H_2_SO_4_ was added, and the OD_450_ was measured using an enzyme-linked immunosorbent assay (ELISA) reader (BMG LABTECH, Ortenberg, Germany). 

### 2.4. Immunization and Sera Collection

Groups of five female six-week-old BALB/c mice (Orient Bio, Seoul, Republic of Korea) were immunized via the subcutaneous route with 20 μg of the recombinant proteins purified by SEC and with endotoxin removed by Triton X-114 (endotoxin level < 1.0 EU/mL). Alum (Imject™ Alum; Thermo Fisher Scientific, Rochester, NY, USA) was mixed in equal volumes to the antigen by gentle inversion, following the manufacturer’s protocol. The immunized mice were administered a booster twice via subcutaneous injection on days 14 and 28. Blood samples were collected through ocular bleeding from the orbital sinuses of the mice on days 27 and 41 after immunization. The collected blood was centrifuged for serum separation at 12,000× *g* for 30 min after stabilization at 4 °C overnight. All animal studies were conducted in accordance with the Institutional Animal Care and Use Committee (IACUC) of Yonsei University (IACUC number, IACUC-A-201911-980-03).

### 2.5. Virus and Serum Antibody Analysis

Dengue virus serotype-1, DenKor-07 (NCCP41507, National Culture Collection for Pathogens, Cheongju, Republic of Korea), was used for this study. The virus was propagated in Vero cells and cultured in minimal essential medium (MEM) at 37 °C. The virus titer was determined by plaque assay using Vero cells. An enzyme-linked immunosorbent assay (ELISA) was performed to measure the immunogenicity and serum levels of IgG1 and IgG2a. Nunc 96-well immunoplates were coated with dengue virus (10^6^ PFU/well) in PBST at 4 °C overnight. Sera from immunized mice underwent two-fold serial dilution with 0.5% BSA in PBST (1/100 to 1/204,800). Anti-mouse IgG1 and IgG2a goat antibodies were used in the ELISA assay.

### 2.6. Neutralization Assay

The plaque reduction neutralization assay (PRNT) was performed using DenKor-07 and Vero cells in six-well plates. Pooled sera from the third immunized mice were diluted in serum-free MEM (1/20 to 1/320) and inactivated by incubation at 56 °C for 30 min. The virus was diluted in serum-free MEM to 100 PFU/well and mixed in equal volumes with the heat-treated sera. The mixtures were incubated at 37 °C for 1 h and applied to Vero cells in six-well plates for inoculation. After inoculation at 37 °C for 2 h, the mixtures were washed out with PBS, and 2% methylcellulose in DMEM was added as an overlay medium. Infected Vero cells in the overlay medium were incubated at 37 °C for 5 days. The number of plaques was counted by a researcher who was blinded to the details of the study. The PRNT_50_ titer was calculated as the highest dilution titer with a 50% reduction in the number of plaques compared with serum-free controls. Statistical analysis of the neutralization titer (NT) was performed by the two-tailed unpaired *t*-test, and the difference was considered statistically significant at *p* < 0.05 (** *p* < 0.01).

## 3. Results

### 3.1. Development of Recombinant Proteins

**Figure 1:**
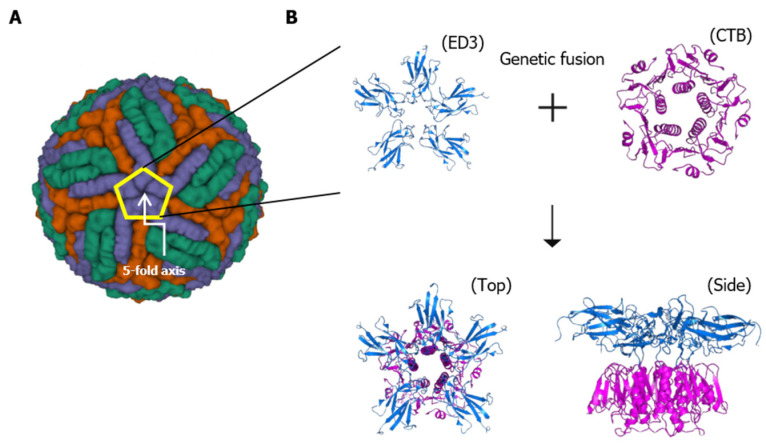
Schematic representation of dengue virion and the architecture of recombinant antigen structures. (**A**) 3D model of the dengue virus structure (PDB ID: 4CCT) and icosahedral five-fold axis. E proteins are shown in blue on the five-fold axis, in green on the two-fold axis, and in orange on the three-fold axis. EDIII is positioned at the end of the E protein along the five-fold axis and converges to a pentameric interface marked in the pentagon. (**B**) Schematic pentamer structure of CTB-DV1EDIII after TEV cleavage. Cholera toxin B subunit (CTB) forms a pentamer, as shown in magenta, and DV1EDIII (blue) constitutes the pentameric interface with assistance of the CTB.

**Figure 2:**
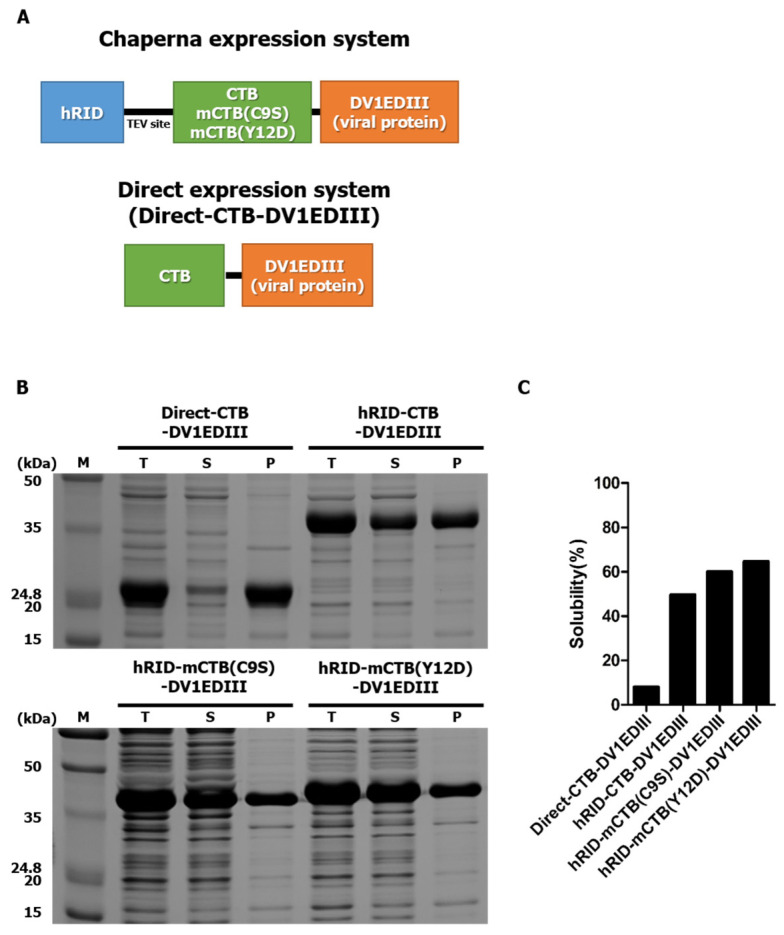
Expression of hRID-CTB-EDIII tri-partite fusion protein in *E. coli*. (**A**) Modular arrangement of hRID-CTB-EDIII (w.t and mutant) and CTB-DV1EDIII without hRID fusion. hRID represents the N-terminal domain of human lysyl-tRNA synthetase, which undergoes structural transition upon binding with tRNA [33]. (**B**) Coomassie blue-stained 12% SDS-PAGE of each construct protein in *E. coli* cell lysates for the solubility test. T, total extract after cell lysis; S, soluble fraction after centrifugation; P, insoluble pellet fraction after centrifugation. (**C**) Solubility index. The relative solubility of each construct was calculated by semi-quantifying the band density by densitometer scanning. hRID represents the N-terminal appendage of human lysyl-tRNA synthetase [28].

Since the DENV EDIII protein induces neutralizing antibodies and converges to the pentameric interface on the viral surface along the icosahedral five-fold axis regardless of conformational changes during cell fusion, we hypothesized that pentameric EDIII could be effective in inducing enhanced neutralizing antibodies against DENV. The dengue virion icosahedral structure of the E protein and the recombinant chimeric proteins emulating the pentameric interface of the virion surface by fusion to CTB are schematically shown in Figure 1A,B, respectively. Two mutant CTB constructs were also generated (Figure 2A), a pentamer assembly defective mutant mCTB(C9S) and a GM1 binding-defective mutant mCTB(Y12D) [30,31]. Other studies have shown that antigens genetically conjugated to CTB result in the misfolding of CTB, which leads to aggregates that prevent pentamerization [25]. Consistent with this finding, CTB-DV1EDIII was predominantly expressed as insoluble inclusion bodies (Figure 2B). To resolve this issue, we decided to employ hRID fusion for solubility enhancement [29,32]. hRID represents the N-terminal domain of human lysyl-tRNA synthetase, which undergoes structural transition upon binding with tRNA [33], executing Chaperna (RNA-dependent chaperone) [28,29] function to the genetically fused target protein. The solubility of CTB-DV1EDIII genetically conjugated with hRID was greatly improved (0% to approximately 50%) (Figure 2C). Similarly, two CTB mutants, hRID-mCTB(C9S)-DV1EDIII and hRID-mCTB(Y12D)-DV1EDIII, were also expressed with high solubility (>60% and >70%, respectively) by fusion to hRID, confirming the solubility/folding enhancement by hRID tags that operate in an RNA-interaction-dependent manner [29,32].

### 3.2. Protein Purification and Pentamer Analysis

**Figure 3:**
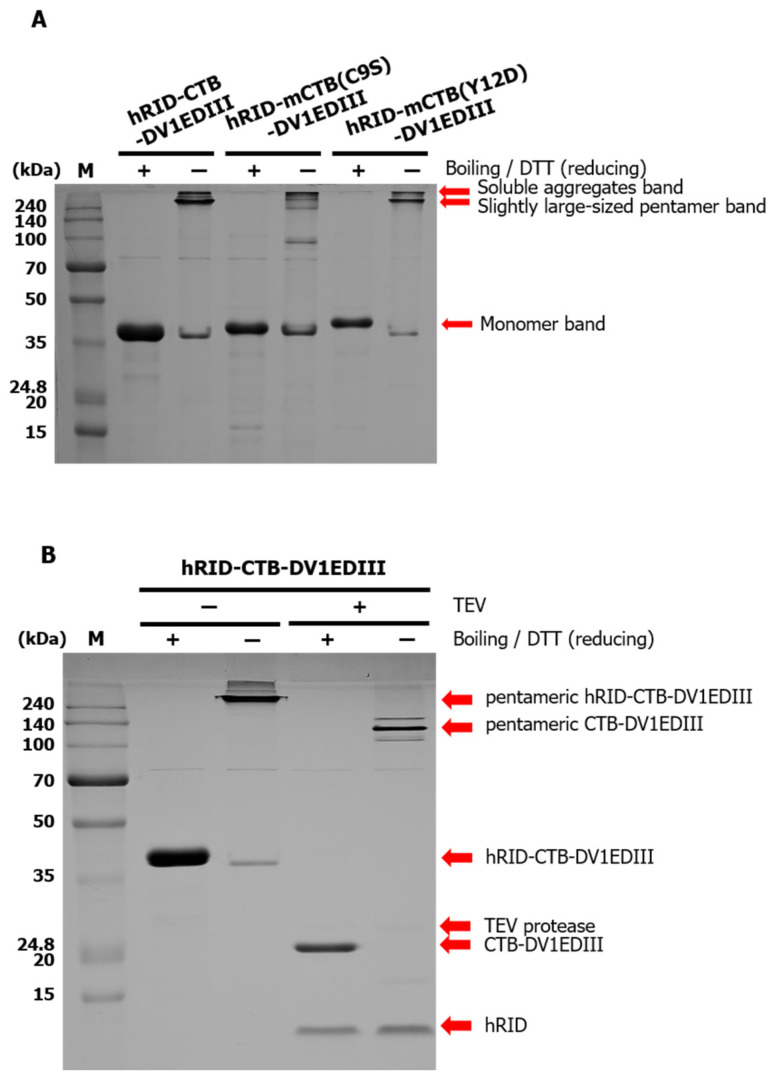
SDS-PAGE analysis of Ni-affinity purified protein. (**A**) Tri-partite fusion protein hRID-CTB-EDIII after Ni-affinity purification under standard conditions (boiling in the presence of DTT) (+) or without boiling and DTT (−). (**B**) hRID cleavage by TEV treatment at 4 °C overnight.

The fusion proteins were purified by Ni-affinity chromatography and semi-quantified by band intensity in SDS-PAGE and compared with known concentrations of BSA. Approximately 14 mg of purified hRID-CTB-DV1EDIII was obtained from 500 mL of *E. coli* culture. To examine the assembly status of CTB fusion proteins, SDS-PAGE was performed without DTT and boiling the protein sample prior to electrophoresis. Under these conditions, bands at the monomer size of hRID-CTB-DV1EDIII and hRID-mCTB(Y12D)-DV1EDIII shifted up to the size where the pentamer was expected. The slight retardation of hRID-CTB-DV1EDIII and hRID-mCTB(Y12D)-DV1EDIII to apparently larger sizes under SDS-PAGE conditions than the expected size (162.5 kDa) is due to the intrinsic disorder of the hRID tag of the Chaperna function [33]. As expected, removal of hRID by TEV cleavage resulted in the expected size of the monomer (22.5 kDa) and pentamer (112.5 kDa) of CTB-DV1EDIII (Figure 3B). However, we failed to observe a pentamer-sized band in hRID-mCTB(C9S)-DV1EDIII, as predicted from the assembly defective mutation [31]. The oversized bands in the lanes (without DTT and boiling) were suspected to be soluble aggregates (Figure 3A).

**Figure 4:**
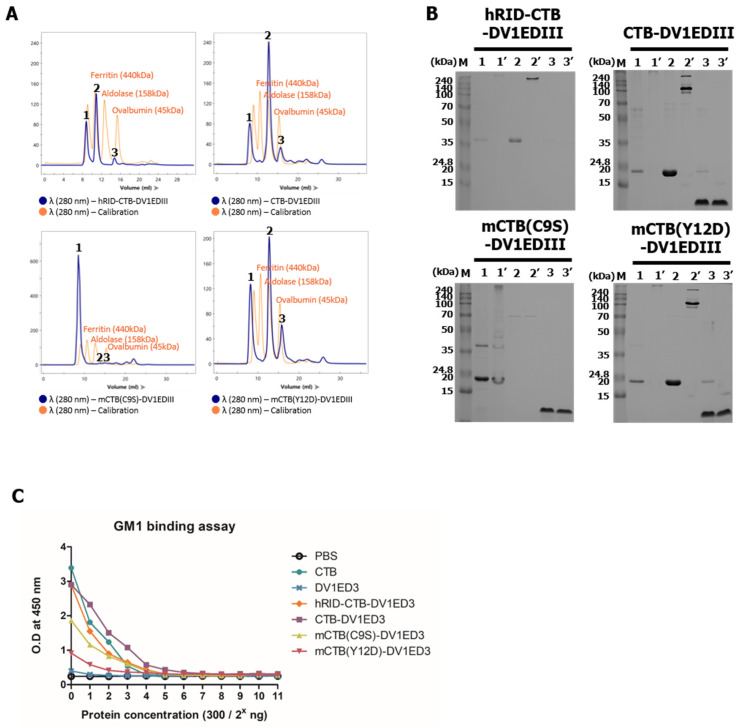
Validation of pentamer assembly. (**A**) SEC analysis. Blue, recombinant proteins; orange, calibration markers, including ferritin (440 kDa), aldolase (158 kDa), and ovalbumin (45 kDa). (**B**) SDS-PAGE analysis of the eluted peak fraction. Lanes 1, 2, and 3 represent samples from peaks 1, 2, and 3, respectively, under reducing conditions (without boiling and DTT); lanes 1′, 2′, and 3′, samples from peaks 1, 2, and 3 under non-reducing conditions. (**C**) GM1 binding ability of recombinant proteins.

In addition to SDS-PAGE, the assembly status of the recombinant proteins was evaluated by size exclusion chromatography (SEC) after TEV cleavage (Figure 4A). The three highest peaks (peaks 1, 2, and 3) were pooled and analyzed by SDS-PAGE (Figure 4B). Peak 1 on the SEC graph, corresponding to the void volume, may represent soluble aggregates, whereas peak 2 represents a properly assembled pentamer. Variable, but in much smaller amounts, monomer-sized bands (peak 3) were also present in all constructs, consistent with the PAGE analysis in Figure 3. As a control, the pentamer-defective mutant mCTB(C9S)-DV1EDIII was eluted at the void volume, confirming soluble aggregates of folding/assembly-defective chimeric proteins (Figure 4A), consistent with the PAGE analyses in Figure 3A. The pentamer was observed as major peak 2 for both CTB-DV1EDIII and mCTB(Y12D)-DV1EDIII, suggesting that the receptor-binding mutation Y12D [30] does not interfere with the folding and assembly of CTB-chimeric proteins. 

As a test for the functional assembly of pentamers, an ELISA assay was performed for the binding with ganglioside GM1 as the main receptor for CTB. Recombinant CTB and DV1EDIII (TEV cleavage of hRID-CTB and hRID-DV1EDIII, respectively) were used as positive and negative controls for GM1 binding, respectively. CTB-DV1EDIII was almost comparable to CTB, suggesting that CTB function was maintained even after chimeric fusion with DV1EDIII. The avidity of CTB-DV1EDIII was slightly higher than that of hRID-CTB-DV1EDIII and was apparently free from structural interference by the disordered hRID domain [33]. In contrast, mCTB(C9S)-DV1EDIII and mCTB(Y12D)-DV1EDIII showed much lower affinity because of the defective assembly of the pentamer (C9S) or lack of affinity to the receptor (Y12D), even after successful assembly into the pentamer (Figure 3A and Figure 4A). The results also showed that hRID functions as a chaperone for the folding and functional assembly of the chimeric antigen into the desired pentameric configuration, overcoming the kinetic trap into insoluble, non-functional aggregates [33] (Figure 2B).

### 3.3. Immunogenicity Study of the CTB Fusion Proteins

**Figure 5:**
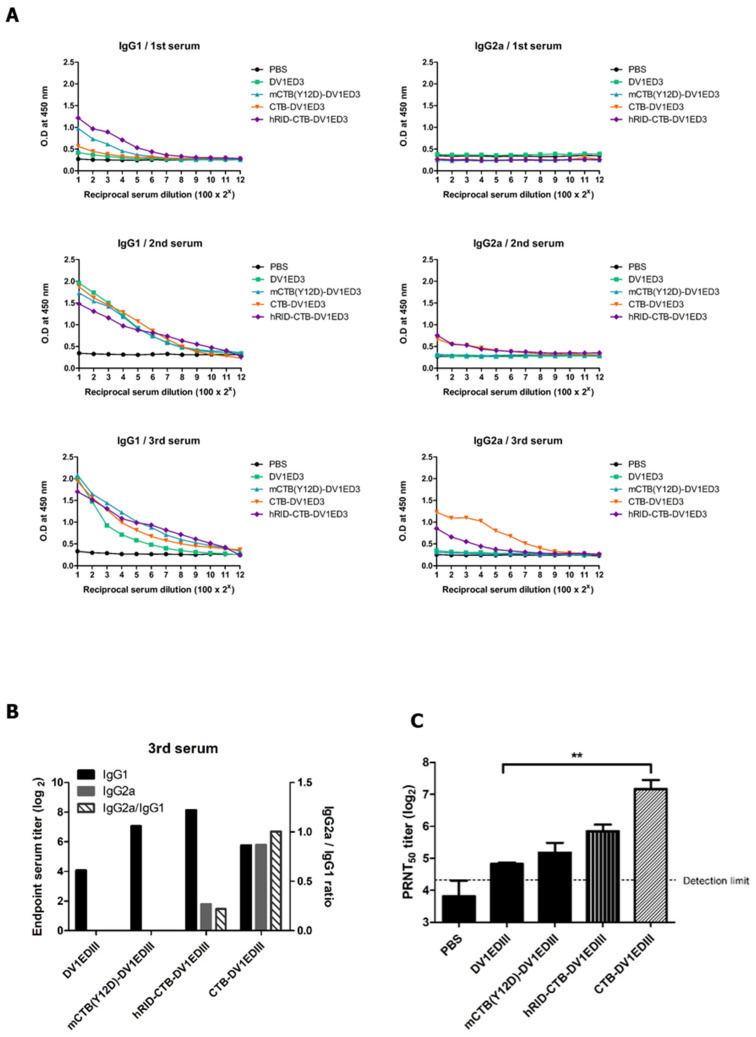
Immunogenicity of recombinant proteins. BALB/c mice (*n* = 5) were immunized with 20 μg of the indicated recombinant proteins via the subcutaneous route three times at two-week intervals. All proteins had endotoxins removed and were formulated with alum before immunization. (**A**) IgG1 and IgG2a antibody responses against the DenKor-07 dengue virus as a coating antigen. The antibodies were two-fold serially diluted with 0.5% BSA in PBST. Data were transformed to their log values. (**B**) Relative ratio of the IgG2a/IgG1 antibody response from the third serum of immunized mice. It was calculated from the log values. (**C**) Neutralizing antibody response against DenKor-07. Data were transformed to their log values, and the standard derivations were expressed as error bars. The detection limit was expressed as a dashed line (20). All data were analyzed by two-tailed unpaired *t*-tests, and the difference was considered statistically significant (** *p* < 0.01).

Groups of five mice were immunized with 20 μg of recombinant proteins mixed with alum subcutaneously on day 0 and boosted with the same dose on days 14 and 28. mCTB(C9S)-DV1EDIII was excluded from the animal experiments because it was identified as a soluble aggregate in SEC and could not be purified as a functional pentamer (Figure 4A,B). IgG1 and IgG2a antibody titers in mouse serum were measured by ELISA using dengue virus as a coating antigen. The serum IgG1 titers for all recombinant proteins, except the mock group, increased after the second immunization. In contrast, IgG2a was elicited only in groups immunized with hRID-CTB-DV1EDIII or CTB-DV1EDIII. CTB-DV1EDIII showed higher IgG2a antibody titers than hRID-CTB-DV1EDIII (Figure 5A), in parallel with a higher avidity to the GM1 receptor (Figure 4C). As an indicator of Th2 versus Th1 responses in mice, the levels of IgG1 and IgG2a and their relative ratios were estimated (Figure 5B). The results show that CTB fusion dramatically increased the IgG2a response (>100 fold), resulting in immune reprogramming into a balanced Th1/Th2 response. The removal of the hRID domain further increased the ratio (approximately fivefold), clearly suggesting that isotype switching in favor of IgG2a is due to the CTB adjuvant function triggered by GM1 binding. To evaluate the NT titer in immunized mouse serum, PRNT was performed using wild-type dengue virus. The NT titers in the third serum of mice immunized with DV1EDIII, mCTB(Y12D)-DV1EDIII, hRID-CTB-DV1EDIII, and CTB-DV1EDIII were 28.3, 37.9, 58.8, and 149.7, respectively. Notably, CTB-DV1EDIII elicited five-fold higher levels of NT than DV1EDIII did. In contrast, mCTB(Y12D)-DV1EDIII, which was defective in GM1 receptor binding, increased the titer only slightly (Figure 5C). The strong correlation between NT titer, GM1 receptor binding, and Th1/Th2 balance suggests that CTB functions as both a built-in adjuvant and a stabilizer of the pentameric configuration of protective epitopes.

## 4. Discussion

Recombinant protein vaccines, especially when combined with bacterial production platforms, are considered suitable for a rapid response to emerging and re-emerging viruses because of their cost-effectiveness and rapid production. As the dengue virus is endemic primarily in developing countries, the economic benefits of a low-cost bacterial production platform are further emphasized. However, a well-known drawback of recombinant protein vaccines is that they induce relatively low immunogenicity compared with live attenuated or inactivated vaccines [34]. The ability to assemble monomeric proteins in higher-order particulate forms increases the chances of antigen presentation to immune cells [19,20,21]. In addition to the self-adjuvanting effect of increasing the size of the antigen, a proper combination with effective adjuvants is instrumental for increasing vaccine efficacy [35,36].

An ideal design of recombinant vaccines would be pivoted by judicious combination of the adjuvant function, for example, by increasing the size of the antigen; by providing a structural scaffold, thus preserving the conformational constraints for protective epitopes; or by serving as a built-in adjuvant [35,37,38]. Here, we aimed to show that such ideal characteristics could be integrated by harnessing CTB as a mucosal adjuvant and structural scaffold. Furthermore, the RNA-based Chaperna function [28,32] was implemented for folding and functional assembly of the chimeric DENV EDIII antigen into a subvirion pentameric configuration, overcoming the kinetic trap into insoluble, non-functional aggregates (Figure 2B). The strong correlation between NT titer, GM1 receptor binding, and IgG isotype switching into a balanced Th1/Th2 response suggests that CTB fusion indeed functions both as a pentameric scaffold to stabilize the five-fold axis interface and as a built-in adjuvant for chimeric antigens.

Many studies have attempted to use CTB as a mucosal adjuvant for vaccine antigens; however, CTB fusion proteins in *E. coli* host systems have been proven to be refractory to soluble expression and assembly [25,39]. For instance, as exemplified by the Japanese encephalitis virus of the same *Flaviviridae* family, this vexing problem has been addressed by the chemical refolding of insoluble inclusion bodies, but in low yields of homo- or hetero-pentameric structures [24,25]. Pivoted by the Chaperna system [28,29,32,33], here, we demonstrate the pentameric assembly of soluble chimeric CTB-DV1EDIII in an *E. coli* expression system as a subvirion recombinant protein approach. The formation of pentameric CTB-DV1EDIII was confirmed using SEC and GM1 binding assays. Soluble expression of CTB-DV1EDIII, which was not possible with conventional methods, was successfully accomplished utilizing Chaperna functions without recourse to the refolding steps. Verified by DENV serotype 1, this platform could be extended to serotypes 2, 3, and 4 (Supplementary Figure S1). Additional studies are warranted to combine all four serotypes into tetravalent-type recombinant vaccines. Remarkably, the yield of hRID-CTB-DV1EDIII after the first purification and dialysis in storage buffer was singularly high (28 mg/L) in a lab-scale flask culture, up to OD_600_ > 1.0. High-density culture can be easily adopted in *E. coli* to further increase the yield [40]. The highest binding affinity for GM1 was observed in CTB-DV1EDIII after the removal of the hRID tag by treatment with TEV protease, probably clearing the steric hindrance imposed by the hRID tag. Mice immunized with antigen protein showed high IgG titers and virus-neutralizing antibodies (Figure 5C). Notably, CTB-DV1EDIII-immunized mice greatly promoted IgG2a, which was not noticeable in the other groups. IgG2a is known to provide antiviral immunity via the influence of cytokines produced by T helper cell 1 (Th1 cell) [41]. The elevated level of IgG2a in a mouse model is considered significant for dengue vaccine efficacy in association with the Th1 subset of CD4+ T cells secreting IFN-γ and TNF [42]. Despite extensive studies, there have been contrasting reports on the role of cholera toxin (CT) in cytokine secretion, and the exact mechanism of its adjuvant activity has not yet been elucidated [43,44]. In addition to the induction of IgG2a associated with cellular immunity, CTB-DV1EDIII induced antibodies with a strong neutralizing effect, probably because of the maintenance of conformational epitope(s) at the pentamer axis. This implies that the adjuvant activity of the CTB works in concert with the proper presentation of a protective antigenic structure to elicit potent neutralizing antibodies. In this study, we validated the pentamer assembly of dengue virus EDIII using CTB and demonstrated its potential in enhancing immune responses. However, recent studies have raised limitations of ED3 in terms of its effectiveness when tested on humans. In order to address this issue, we intend to further study whether this platform could be extended to the whole E protein and various combinations of NS proteins, enabling the induction of additional T cell immune response in future studies. We are also contemplating future studies to explore various vaccine administration routes, such as oral administration, leveraging the mucosal adjuvant properties of CTB. Further studies should also confirm the actual defensive effect against dengue virus challenges in animal models.

A major challenge for dengue vaccines is to provide balanced protection against the four serotypes in the form of a tetravalent vaccine [45]. The epitopes of several potent neutralizing human mAbs have been identified by cryo-EM studies; they recognized conformational and discontinuous epitopes involving different domains, such as the E dimer epitope, hinge region, or residues involving domains I, II, and III from different monomers [46]. It has been proven that it is more difficult to develop a tetravalent vaccine that protects against each of the four serotypes than to develop monovalent vaccines that protect against corresponding serotypes due to antibody-dependent enhancement (ADE) against different serotypes [14,47]. Further studies are therefore required to determine whether the present strategy of utilizing CTB could efficiently control the balance of the immune response induced by antigens of each serotype to overcome this challenge. This study initially targeted one specific serotype (DENV1), and future studies on tetravalent proteins covering all four serotypes would confirm potential safety issues related to cross-reactivity into ADE. Our next goal is to analyze the neutralizing effect against each serotype and to develop a final tetravalent vaccine. We have initiated the production of antigenic proteins for dengue virus serotypes 2, 3, and 4 in subsequent studies (Figure S1). Subsequently, biological functionality testing and measurement of neutralizing antibody titers through animal experiments are planned for these antigens. The design principle of the pentamer-based subvirion recombinant protein approach, as proven with dengue—CTB as a structural scaffold, a built-in adjuvant, and a multimeric assembly of protective antigens—can be generally applied to flaviviruses comprising dengue, JEV, Zika, and West Nile viruses, as well as other viruses with icosahedral assemblies. 

## 5. Conclusions

In summary, we successfully generated soluble pentameric CTB-DV1EDIII with high yield using an *E. coli* expression system with the aid of our RNA-based chaperone (Chaperna) system [28]. We confirmed the pentameric assembly and assessed the biological activity, including GM1 binding affinity, of CTB-DV1EDIII through size-exclusion chromatography (SEC), GM1 binding assays, and transmission electron microscopy (TEM) (Figure S2). Furthermore, immunization of mice with CTB-DV1EDIII resulted in the induction of IgG2a antibodies. Sera from CTB-DV1EDIII-immunized mice exhibited higher neutralizing antibody titers when compared to sera from mice immunized with DV1EDIII or mCTB(Y12D)-DV1EDIII. Our study presents a highly soluble bacterial production platform for immune-enhanced dengue antigens achieved by fusing with CTB, serving as both a pentameric scaffold and built-in adjuvant.

## Data Availability

All datasets in this study are available from the corresponding author on reasonable request.

## Supplementary Materials

The following supporting information can be downloaded at https://www.mdpi.com/article/10.3390/vaccines12010092/s1: Figure S1: SDS-PAGE analysis of EDIII expressions of dengue virus serotypes 2, 3, and 4. Coomassie blue-stained 12% SDS-PAGE of each construct protein in *E. coli* cell lysates for the solubility test. T, total extract after cell lysis; S, soluble fraction after centrifugation; P, insoluble pellet fraction after centrifugation. Figure S2: TEM analysis of CTB-DV1ED3 and mCTB(C9S)-DV1ED3. The protein samples were placed onto a TEM grid (SPL), followed by negative staining with a 1% phosphotungstic acid (Sigma). Subsequently, an image was captured using a JEM-F200 operating at an accelerated voltage of 200 kV. CTB-DV1ED3 imaged at a scale of 100 nm, illustrating the formation of pentamers. mCTB(C9S)-DV1ED3 imaged at a scale of 20 μm, revealing the presence of large aggregates.
