# Supplementary figures and images for: Harnessing Pentameric Scaffold of Cholera Toxin B (CTB) for Design of Subvirion Recombinant Dengue Virus Vaccine
Source: Vaccines (Basel). 2024 Jan 17;12(1):92. doi: 10.3390/vaccines12010092 (PMC10819241; doi:10.3390/vaccines12010092)

Supplementary Figure S1

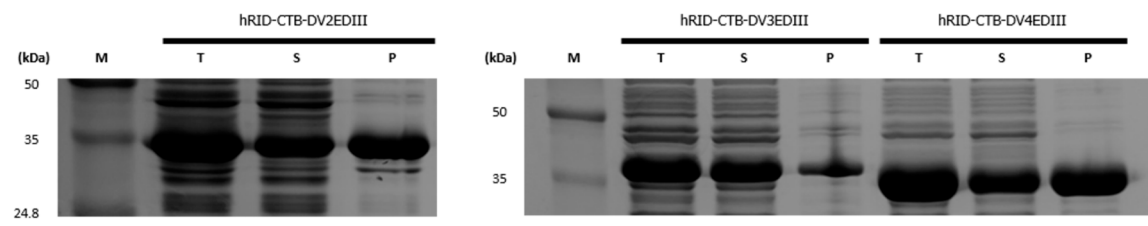

Supplementary Figure S2

**CTB-DV1EDIII**

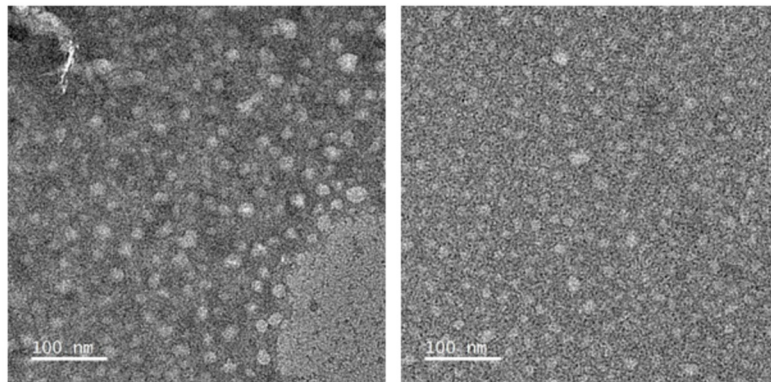

**mCTB(C9S)-DV1EDIII**

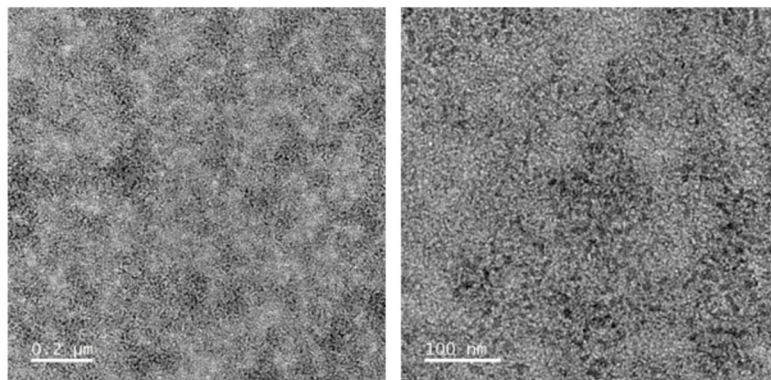

Supplement: Supplementary file 1 [file vaccines-12-00092-s001.zip › vaccines-2796796-supplementary.pdf]
